# Seroprevalence of hepatitis E virus infection in blood donors from Piauí State, Northeast Brazil

**DOI:** 10.1016/j.bjid.2024.104466

**Published:** 2024-11-26

**Authors:** João Paulo da Silva-Sampaio, Raniela Borges Sinimbu, Julia Trece Marques, Abilio Francisco de Oliveira Neto, Livia Melo Villar

**Affiliations:** a Fiocruz Piauí, Teresina, PI, Brazil; b Fundação Oswaldo Cruz, Instituto Oswaldo Cruz, Laboratório de Hepatites Virais, Rio de Janeiro, RJ, Brazil; c Centro de Hematologia e Hemoterapia do Estado do Piauí, Teresina, PI, Brazil

**Keywords:** Hepatitis E virus, Blood donors, Epidemiology

## Abstract

A retrospective and cross-sectional study was carried out on blood donors from Piauí State located at Northeastern Brazil to evaluate the prevalence of Hepatitis E Virus (HEV) infection. Serum samples were tested for anti-HEV IgG and IgM using electrochemiluminescence and HEV RNA was tested using real time PCR. A total of 890 individuals were included with median age of 33.4 years and most of them were male and lived at Mid-Northern region of the State. Prevalences of anti-HEV IgG and IgM were 1.35 % and 0.11 %, respectively. None HEV-RNA was detected. This study demonstrated low prevalence of HEV infection in blood donors in this region.

According to the World Health Organization (WHO), an estimated 20 million people are infected with the Hepatitis E Virus (HEV) annually, with around 3.3 million symptomatic cases. In 2015 alone, approximately 44,000 individuals died as a result of HEV infection.^1^

HEV antibodies was detected in serum samples from acute hepatitis cases in Latin America.^2^^,^^3^ Although Brazil is not endemic for hepatitis E, antibodies specific to HEV (anti-HEV) were found in different population groups. Prevalence varies from 0.15 % to 19.5 % among indigenous population, recyclable waste pickers, immigrants and refugees, homeless individuals, lesbian, gay, bisexual, and transexual individuals, crack users, residents in a low-income area, cirrhotic and liver transplant patients and blood donors from different geographic regions of Brazil.^3^, ^4^, ^5^, ^6^ These studies were conducted in all regions of Brazil, but few studies were performed in Northeast Brazil.^7^^,^^8^

HEV is not routinely investigated in Brazil, even in cases of unexplained elevation of liver enzymes or acute hepatitis, and only few laboratories perform anti-HEV tests. Few recent studies have been conducted to evaluate anti-HEV positivity in northeastern Brazil, which is considered a high prevalence setting for hepatitis A, which is also transmitted by the fecal-oral route. This study aims to assess the prevalence of antibodies against hepatitis E in blood donors from the state of Piauí (Northeast Brazil), which is divided into four health macro-regions that are highly complex healthcare references (Mid-Northern, Coastal, Semi-arid and Cerrado).^9^

A retrospective and cross-sectional study was carried out involving 890 blood donors who donated serum samples in October at 2020 year in from the Piauí State's Hematology and Hemotherapy Center (HEMOPI), Northeastern Brazil. During this month, 3764 samples were donated, and sample size was calculated as sufficient to successfully estimate HEV seroprevalence of approximately 10 % in the city's population with a 95 % Confidence Interval (95 % CI).

The study was approved by the Human Research Ethics Committee of Oswaldo Cruz Institute under protocol number 5.032.276 and CAAE 41,104,620.4.0000.5248. Data regarding date of birth, location of residence and sex were obtained in physical records.

The serum samples were tested using fully automated chemiluminescent immunoassays for the detection of anti-HEV IgG and IgM (Liaison® Murex Anti-HEV IgG and IgM assays, DiaSorin, Saluggia, Italy), with 97.92 % and 98.44 % sensitivity and 99.22 % and 97.93 % specificity for anti-HEV IgG and IgM assays, respectively. Both assays were coated with genotype 1 and genotype 3 ORF2 antigens, and 100 µL of serum samples were used in each assay. The Quantitative Liaison® Murex HEV IgG assay measures the intensity of the luminescence. This intensity is proportional to the concentration of HEV IgG in the serum sample. Test results are automatically calculated by the instrument and are expressed in IU/mL. IgG values below the cut-off value (0.3 IU/mL) are considered to be negative. For the Liaison® Murex HEV IgM qualitative assay, the values obtained from this assay are automatically calculated by the instrument and expressed as an index. Samples with an IgM index value below the threshold (set at 1.00) were considered negative.

Hepatitis E virus ribonucleic acid (HEV RNA) was extracted from 140 μL serum using a QiaAmp viral RNA kit (Qiagen, Hilden, Germany) according to the manufacturer's instructions in anti-HEV IgG/IgM positive samples. Superscript III (Invitrogen, Carlsbad, CA, USA) was used for Reverse Transcription (RT) of RNA at 50 °C.^10^ Real-time PCR was performed to detect (limit of four copies of HEV RNA per reaction) and quantify HEV RNA using TaqMan real-time PCR technology.^11^

Data were analyzed using IBM Statistical Package for the Social Sciences® (IBM SPSS statistic for windows version 26.0). Relative and absolute frequencies and measures of central tendency (median and interquartile range) were calculated for nominal variables and for continuous variables, respectively. A 95 % Confidence Interval (95 % CI) was used to estimate prevalence.

A total of 890 individuals were included in this study. As shown in Table 1, the median age of the study population was 32 years (Interquartile Range [IQR 14-years]) and most of them were male (62.6 %) and lived at Mid-Northern region of Piaui State (73.3 %).

**Table 1:** Socio demographic characteristics of 890 blood donors included in the study.

Characteristic	n (%)	95 % CI
*Gender*		
Male	557 (62.6)	(59.4‒65.7)
Female	333 (37.4)	(34.3‒40.6)
Age (median; IQR)		
16‒29 (24; 5)	350 (39.3)	(36.2‒42.6)
30‒44 (36; 7)	410 (46.1)	(42.8‒49.4)
45‒59 (51; 7)	124 (13.9)	(11.8‒16.3)
60–69 (62; 1)	6 (0.7)	(0.3‒1.4)
*Health Macro-regions*		
Cerrado	67 (7.5)	(5.9‒9.4)
Coastal	76 (8.5)	(6.8‒10.5)
Mid-Northern	652 (73.3)	(70.3‒76.1)
Semi-arid	95 (10.7)	(8.8‒12.8)

95 % CI, Confidence Interval for proportion; IQR, Interquartile Range.

Among the 890 participants tested for anti-HEV IgM and IgG markers, twelve individuals were anti-HEV IgG-positive 1.35 % (95 % CI 0.77–2.34), while one was also positive for anti-HEV IgM 0.11 % (95 % CI 0.02–0.63) but negative for HEV-RNA.

Anti- HEV IgG was more prevalent in males (8/12), with ages equal or >30 years (9/12) and living in Mid-Northern Macro region of Piaui State (11/12). These prevalences did not presented statistical significance according to these variables as shown in Table 2.

**Table 2:** Univariate analysis of sociodemographic data of blood donors screened in the study.

Characteristic	Anti-HEV IgG positive	Anti-HEV IgG negative	OR (95 % CI)	p^a^
	*n* = 12 (%)	*n* = 878 (%)		
***Gender***				1.00
Male	8/12 (66.7)	557/878 (63.4)	1.199 (0.358 – 4.012)	‒
Female	4/12 (33.3)	321/878 (36.6)	‒	‒
***Age group***				0.38
< 30 years	3/12 (25.0)	350/878 (39.8)	0.51 (0.13 – 1.89)	‒
≥ 30 years	9/12 (75.0)	528/878 (60.2)	‒	‒
***Health Macro regions***				0.19
Mid-Northern	11/12 (91.7)	641/878 (73.0)	0.24 (0.03 – 1.91)	‒
Other regions	1/12 (8.3)	237/878 (27.0)	‒	‒

a Fisher Exact Test. OR, Odds Ratio; CI, Confidence Interval.

In the Northeast region of Brazil, limited data are available on the prevalence of HEV infection. In the present study, it was found seroprevalence of 1.35 % of anti-HEV IgG among blood donors in Piaui State which is higher than observed in recent studies in Pernambuco State (0.9 %)^8^ and in the Amazon State (0.44 %)^12^ located in the northeast and north regions of Brazil, respectively. On the other hand, high prevalences of anti-HEV IgG was found in blood donors from Southeast (9.8 %)^13^ and Mid-West (6.4 %)^14^ regions of Brazil. Even higher prevalence was observed among general population in South Brazil (57.4 %)^15^ what was probably associated to the presence of the virus on the environment and/or products of animal origin for human consumption.

Such discrepancies may be related to different regional characteristics; performance of the serological tests used; sample size and housing infrastructure, drinking water and sewage. Furthermore, scientific evidence has demonstrated that socioeconomic and cultural aspects, such as eating raw or poorly cooked pork, can also influence the seroprevalence of this infection,^16^ since probable zoonotic transmission of HEV has been demonstrated in Brazil.^17^

In the present study, one individual presented anti-HEV IgM representing a recent infection, but it was HEV RNA negative demonstrating that this is not active case of infection. HEV RNA prevalence was higher among blood donors from France (0.045 %)^18^ and Spain (0.03 %).^19^

Anti-HEV IgG positivity was higher in individuals aging >30 years which was also observed for blood donors from South Brazil.^6^ This demonstrates that exposure to HEV probably occurs more frequently in adults. We also observed that males presented high frequency of anti-HEV IgG as the same as observed in previous studies in blood donors.^6^^,^^8^ Furthermore, the Mid-Northern region, where the capital of Piauí is located, had the highest number of anti-HEV cases in blood donors among the macro-regions studied. This may be due to the fact that it is the most populous region in the State of Piauí and accounts for >50 % of the State's blood supply.

The present study has some limitations, such as the small sample size and the lack of information related to associated factors such as sewage availability, data on contact with animals, as well as eating habits and other risk behaviors. Although these limitations, giving the fact that little is known about the HEV epidemiology in Northeast Brazil, this is the first study to evaluate the seroprevalence of HEV among blood donors in Piauí State giving new information about this infection in Northeast region of Brazil.

## Conflicts of interest

The authors declare no conflicts of interest.
